# Perceived influence of commercial milk formula labelling on mothers’ feeding choices in Great Britain: a qualitative study

**DOI:** 10.1136/archdischild-2023-325767

**Published:** 2023-08-04

**Authors:** Rana Conway, Isabel Ritchie, Sara Esser, Andrew Steptoe, Andrea D Smith, Clare Llewellyn

**Affiliations:** 1 Research Department of Behavioural Science and Health, University College London, London, UK; 2 MRC Epidemiology Unit, University of Cambridge, Cambridge, UK

**Keywords:** Child Health, Paediatrics, Psychology, Qualitative research

## Abstract

**Objective:**

To understand how mothers use commercial milk formula (CMF) labels to inform their feeding choices and explore mothers’ understanding of differences between CMF products.

**Design:**

Qualitative study with recruitment via social media. Online semistructured interviews, including a product mapping exercise and thematic analysis.

**Participants:**

Mothers (n=25) using CMF for children <3 years living in Great Britain (GB).

**Results:**

Mothers were drawn to brands they recognised from years of exposure to CMF advertising. CMF products were assumed to vary according to brand and stage, but participants found on-pack information did not explain how. This added to anxiety about choosing ‘the best one’ and mothers would have liked guidance from healthcare professionals (HCPs). Wide availability of CMF for older infants and children, and on-pack messaging suggesting progression from one product to the next, led many to believe these products were necessary. There was confusion over the appropriate use of specialist products. While mothers rarely mentioned on-pack health and nutrition claims, they were attracted to the overall appearance of packs and messaging relating to science, research and nature. References to breast milk and a logo perceived to represent a breastfeeding mother were taken as indicators of closer similarity to breast milk.

**Conclusions:**

CMF legislation in GB should be updated to restrict brand advertising and the use of on-pack text and images that mothers perceive as indicating products have a closer similarity to breast milk. Greater input from HCPs was desired by new mothers and would support them to make more informed choices about CMF.

**WHAT IS ALREADY KNOWN ON THIS TOPIC**

- Sophisticated and well-funded marketing strategies are used to sell commercial milk formula (CMF).
- Caregivers do not find nutrition and health information on CMF packs helpful when choosing between different products.

**WHAT THIS STUDY ADDS**

- On-pack branding was key to determining choice, as brand trust had developed over years of exposure to advertising.
- On-pack messaging, including imagery, was understood by mothers as indicating certain products were superior or more similar to breast milk than others.
- Mothers indicated they would value more support and guidance from healthcare professionals (HCPs) when choosing CMF.

**HOW THIS STUDY MIGHT AFFECT RESEARCH, PRACTICE OR POLICY**

- Legislation is needed to control CMF brand promotion, and the use of messaging perceived to imply some products are superior or more similar to breastmilk.

## Introduction

Great Britain (GB) reports one of the lowest breast feeding rates worldwide despite national government advice to breast feed exclusively for the first 6 months.^1 2^ Health service estimates suggest only 48% of infants in England are breast fed to any extent at 6–8 weeks,^3^ with higher figures reported in Scotland (55%) and lower rates in Wales (37%) at 6 weeks.^4 5^

**Figure 1:**
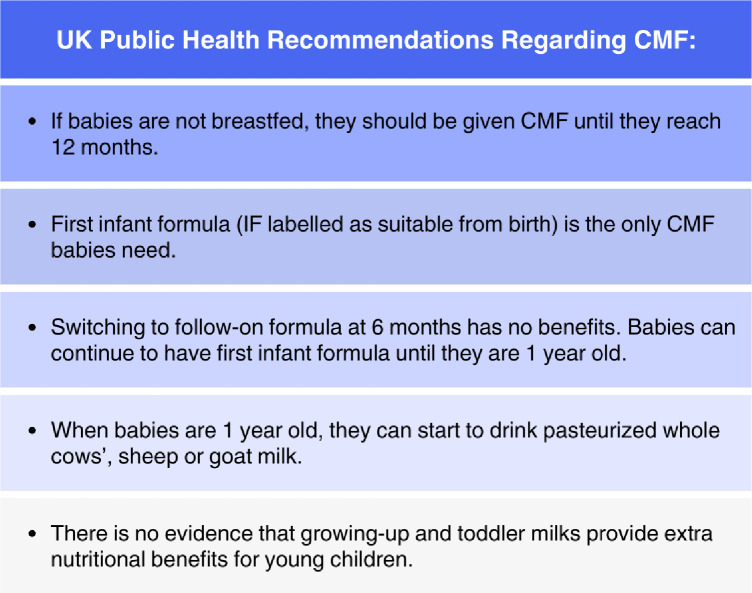
UK Public Health recommendations regarding commercial milk formula (CMF). Adapted from NHS.^33^

Commercial milk formula (CMF) is a vital source of nutrition for infants who are not breast fed (figure 1). Therefore, European Union (EU) legislation, which was adopted by all three countries in GB following EU exit, ensures all infant formula (IF) for infants aged 0–6 months have a standardised nutrient content, likewise follow-on formula (FOF) marketed as ‘stage 2’ for infants aged 6–12 months has a standardised content.^6 7^ Infant feeding methods are determined by a range of factors, and the legislation is also intended to help caregivers identify CMF that is suitable from birth, and protect the public from undue commercial influence so as not to discourage breast feeding.^6 7^ For example, direct-to-consumer advertising of IF is prohibited. However, there is concern that parents still see advertisements for FOF and growing-up formula (GUF) marketed as ‘stage 3’ for toddlers from 12 months in almost identical packaging to IF.^8–11^ Mothers have even reported ‘seeing’ IF advertisements despite their legal inexistence.^12 13^ There is also increasing concern over the pervasive marketing used to generate product demand and sales for more expensive products.^8 12 14 15^ Common practices include focusing attention on breast feeding problems, pitching CMF as the solution to night waking and other common infant behaviours, overstating similarities with breast milk and tapping into new parents’ anxieties and aspirations.^8 16–18^

WHO has highlighted the challenges of tackling increasing digital marketing of CMF via apps, virtual ‘baby clubs’, social media influencers and platforms such as Facebook.^19^ The focus of this paper is on-pack labelling, which, by contrast, is easier to regulate and legislation is easier to enforce. Caregivers in Australia report not finding on-pack nutrition and health information helpful when trying to identify differences between products.^20^ However, little is understood (by those outside the CMF industry) about caregivers’ overall experience and interpretation of packaging. Our previous research found labels on CMF sold in GB often contravene government guidance.^11^ This study aims to: (1) understand how mothers use CMF labels to inform their feeding choices; and (2) explore mothers’ beliefs about differences between CMF products.

## Methods

### Participant recruitment

Eligibility criteria included: >18 years old; parent or main caregiver of child <3 years old currently consuming CMF; GB resident; and able to speak English. Participants were recruited through social media (primarily Facebook) to discuss infant feeding choices. Demographic information was collected with an online questionnaire. Quota sampling was used to select participants according to a composite socioeconomic status score^21^ (low, medium, high) and child’s age (<6 months, 6–11 months, 12–35 months). Purposive sampling was further employed to select participants from diverse households in terms of ethnicity and household composition (single or dual parent households, number of children).^22^ A sample size of 16–24 was considered sufficient to reach a richly textured understanding of the issues.^23^ Participants received a £25 shopping voucher.

### Interviews

One-to-one semistructured interviews were conducted between September and December 2021 via Microsoft Teams or Zoom. A four-part interview schedule was used (online supplemental appendix). Part A explored participants’ feeding experiences. Part B involved product mapping, with participants arranging 11 formula products into groups according to perceived similarity, using Microsoft Whiteboard. In part C, participants viewed and discussed all sides of a single formula pack. Part D focused on infant foods and is not presented in this paper.

10.1136/archdischild-2023-325767.supp1 Supplementary data

Interviews were conducted by RC (n=22), a mother of teenage children, and SE (n=3) who does not have children. Both interviewers are female health researchers, of white European background, unknown to participants and introduced as ‘researchers’. Interviewers made brief interview notes and interviews were audio recorded and transcribed verbatim. Participants did not review transcripts nor findings.

### Analysis

Thematic analysis taking an interpretivist approach was used, which acknowledges the role of researchers in interpreting participant experiences.^24 25^ RC listened to all recordings, read transcripts and generated an initial thematic framework using both deductive and inductive methods, informed by prior knowledge of CMF regulations and findings from quantitative analysis of on-pack labels.^11^ IR then familiarised herself with transcripts and coded them with NVivo software, using RC’s initial framework. RC and IR used an iterative process, adapting codes and redefining themes to develop a more comprehensive coding framework, which IR used to recode transcripts and finalise the code book.

While conducting and analysing interviews, researchers reflected on, and discussed, their personal experiences and thoughts about infant feeding. The Consolidated Criteria for Reporting Qualitative Research checklist was adhered to.

## Results

Twenty-five mothers were interviewed once (mean duration: 54 min (SD 6)) (table 1). Interviewers agreed that sufficient depth of understanding had been reached. Three themes and 10 subthemes were generated, which are summarised with quotes in table 2.

**Table 1:** Interview participants

	Age group of participants’ child consuming CMF			
	Less than 6 months (n=8)	6–11 months (n=9)	12–35 months (n=8)	Total (n=25)
Age of participant in years, mean (SD)	29.6 (5.1)	29.4 (3.5)	30.7 (6.9)	29.9 (5.1)
Number of children, n (%)				
One	5 (63)	6 (67)	6 (75)	17 (68)
Two	1 (13)	3 (33)	1 (13)	5 (20)
Three or more	2 (25)	0	1 (13)	3 (12)
SES, n (%)^21^				
Low	2 (25)	4 (44)	3 (38)	9 (36)
Medium	3 (38)	3 (33)	2 (25)	8 (32)
High	3 (38)	2 (22)	3 (38)	8 (32)
Annual household income below £30 000, n (%)	2 (25)	3 (33)	3 (38)	8 (32)
Highest level of education, n (%)				
GCSE or equivalent	3 (38)	4 (44)	1 (13)	8 (32)
A-level or equivalent	0	3 (33)	1 (13)	4 (17)
Degree or higher	5 (63)	2 (20)	6 (75)	13 (52)
Location, n (%)				
England	6 (75)	6 (67)	3 (38)	15 (60)
Scotland	2 (25)	2 (22)	4 (50)	8 (32)
Wales	0	1 (11)	1 (13)	2 (8)
Ethnicity				
White	6 (75)	8 (89)	4 (50)	18 (72)
Black Caribbean	1 (13)	0	0	1 (4)
Asian	0	1 (11)	2 (25)	3 (12)
Mixed white/black Caribbean	0	0	2 (25)	2 (8)
Mixed white/not listed	1 (13)	0	0	1 (4)
Single parent, n (%)	2 (25)	2 (22)	2 (25)	6 (24)
Mixed CMF and breast feeding, n (%)	2 (25)	3 (33)	2 (25)	7 (28)
Age of infant in months, mean (SD)	2.5 (1.6)	8.2 (1.0)	20.6 (6.3)	10.4 (8.4)
Female infant, n (%)	6 (75)	4 (44)	4 (50)	14 (56)

CMF, commercial milk formula; GCSE, General Certificate of Secondary Education; SES, socioeconomic status based on composite score.

**Table 2:** Themes and subthemes

**Theme 1: It’s all about the brand.**	
1.1 Importance of choosing a trusted brand.	*‘To be honest, I think most of it just came up from actually what I'd seen over just my lifetime of adverts actually and TV things, and what became like a familiar sort of brand that you'd heard of. So, I'd seen a lot of [brand X] adverts, I’d seen a lot of I think it’s the [Brand Y] one as well, I’ve seen quite a few adverts…It’s actually literally just been from advertising, sort of, I guess a trusted brand name that you've kind of heard, especially being a new mum, you want something you know.’* (P14, 2 months)
1.2 Caution over swapping brands.	*‘If she wasn’t happy with it then, yes I would have changed but she actually likes it. She is very, very healthy so I just stayed with the [Brand X]*.*’* (P01, 27 months)
1.3 Information sources for brand choice.	*‘It was more friends and family rather than the midwife with what formula feed is best….They were saying [Brand X] was best with less chance of getting colic for the babies, so in the end they said [Brand X] has got the same breast nutritions in it too.’* (P25, 0.5 months)
1.4 Identifying the best product.	*‘it definitely appeased something in me that I was getting [Brand X]…. I thought in my head at that time, well, if I was going to formula feed, then I had the best on the market and that was it. But, if I wasn’t a mum at 4am in [supermarket], I would tell myself that is absolute nonsense, and there’s probably little to no difference in any of them’*. (P09, 8 months)
**Theme 2: Formula stages exist for a reason.**	
2.1 It’s best to follow the labels.	*‘with my other two I put them onto cows’ milk at one. But I know now [Brand X] has stage three and it’s got more vitamins in it. So, depending on how money is I might, we might carry on with stage three but it is so expensive [laughs] not going to lie.’* (P15, 3 months) *‘I find that information quite confusing actually because some of the products market formula milk that you’re meant to start at 6 months and then some of the guidance is actually you’re weaning solids at 6 months but keeping them on the same milk. So yes, no, we need to do some research on that, I need some more guidance on that.’* (P17, 4 months)
2.2 Different stages are surely different somehow.	*‘I don't know what the difference is with the toddler milks to be honest. But for me, because I'm an anxious person, if it said don’t use it, use this after six months and use this after a year, I would go by what it said, rather than using my own judgement type thing.’* (P18, 15 months) *‘I asked the health visitor and she just said, “Oh keep him on stage one, that’s fine.” But then I’m like, that’s all well and good but why is there then a stage two? She didn’t explain.’* (P23, 8 months)
2.3 Confusion over specialist formula.	*'the [Brand X] Comfort – what does that one say on it? “For colic”. Okay.’* (P12, 10 months) *‘so the Comfort I know is good if they suffer with constipation or reflux…and obviously [Brand X] Lactose Free would be good if you thought they had an allergy.’* (P08, 8 months) *‘[Brand X] Comfort is like the one for keeping the babies fuller for longer.’* (P13, 8 months)
**Theme 3: Presentation matters.**	
3.1 Overall appearance tells a story.	*‘you want to make sure they are getting the best, I suppose and you want to have some faith in the product. I don’t think [Brand X] product really screams─ I don’t know. I don’t know. I just don’t like it. I look at it and I think it’s childish.’* (P20, 3 months)
3.2 Expensive-looking products are better, aren’t they?	*‘It is up to you, I suppose, to do your research. Because if they are all the same, then why don’t we just go for the cheapest? That’s the question. We are programmed to think one is better than the other and they are probably not, are they?’* (P12, 10 months)
3.3 Messages that sell.	*‘to me, “leading baby nutrition research for over 100 years” is way more important, and way more grabbing and confidence building. There’s certain things you would just expect there to be and Omega 3 and 6 is probably one of the things that I would just not be shocked is in there, that’s great. What would sway me more to buy this is definitely if they’re leaders for 100 years.’* (P09, 8 months) *‘for me that was something I would look quite closely, as a breastfeeding mum, on the packaging if it says it’s “breastfeeding friendly”, it tells me that it’s really close, in like to likeness to breastmilk.’* (P12, 10 months) [‘Breastmilk substitute’ was written on product viewed] *‘[Brand X logo] stands out because it’s very simple but it proper stands out. It looks like a mother but then her breast, so it’s showing it’s similar to breast milk.’* (P25, 0.5 months) *‘the M in the middle kind of looks like a breast feeding mum so I kind of look at that and think oh maybe it’s good because it’s kind of got that breast feeding association with it so you are giving a pure feed kind of, that’s kind of what I get from seeing that.’* (P10, 9 months)

P refers to participant number; months refers to the age of child consuming commercial milk formula (CMF).

### Theme 1. It’s all about the brand

CMF brand was seen as the chief distinguishing feature of packs. Mothers consistently described brands as having distinct identities and brand trust was key to formula choice (theme 1.1). Prior to choosing a CMF they were already very familiar with the market leader brands, which they viewed as a ‘safe choice’ as they perceived them as having ‘done their research’. Mothers recalled years of exposure to television advertisements plus recent proactive information seeking from friends, family, brand websites and social media forums.

Swapping brands was considered carefully. Mothers not experiencing feeding problems viewed it as risky, and those with problems viewed swapping as a possible solution (theme 1.2). One mother, whose baby was constipated, described switching to an alternative brand after ‘googling’ the problem and finding overwhelming recommendations for that new brand from mums in the brand’s Facebook group. Interestingly, two mothers who had to switch brands due to availability issues reported their babies not noticing.

Many mothers would have liked more feeding support from healthcare professionals (HCPs), and some felt this abruptly stopped when they were no longer viewed as potential breast feeders. Several mothers recalled asking a midwife or health visitor to recommend a brand and being informed that recommendations were not permitted. This caused frustration and resulted in them turning to alternative information sources, including friends and brand websites (theme 1.3). Mothers felt overwhelmed by the large number of formula products available and many recalled scrutinising labels and brand websites to compare products and identify ‘the best one’ (theme 1.4). Reading or being told by an HCP that all products were the same gave some the confidence to choose the cheapest brand, although others still felt unsure about doing this. Some mothers recognised an element of inconsistency in their thoughts and beliefs about choice of CMF, suspecting marketing may be the only real difference.

### Theme 2. Formula stages exist for a reason

While brand was felt to be the most important feature used to distinguish one formula pack from another, stage was also vital. The importance of starting with products labelled as suitable ‘from birth’ was universal but beliefs about how stages differed, and whether FOF was needed, varied. First-time mothers often felt it was best to follow the guidance on CMF labels (theme 2.1). They discussed babies ‘progressing’ from one formula stage to the next, viewed formula companies as experts and believed FOF and GUF would not exist if they were not necessary. However, one mother described FOF as ‘just marketing fluff’. Individuals who confidently stated ‘all they need is first milk’ often recounted a single conversation with a health visitor or friend, or reading a particular article. Many worried about making the right decision over switching to FOF or not. Likewise, choosing cow’s milk over GUF at 12 months caused concern for some, who had heard cow’s milk might cause digestive problems or that you could never be sure a toddler’s diet provided all the nutrients GUF supplied. Several experienced mothers felt confident GUF was unnecessary or an expensive gimmick.

Even those convinced that IF and FOF were different struggled to pinpoint how, despite having previously scrutinised ingredient and nutrient panels on packs (theme 2.2). Ideas included IF being softer, lighter, easier to digest or more similar to breast milk, which was seen as an explanation for labelling it as a ‘breastmilk substitute’. Some suggested IF was more tightly regulated, higher in calories or higher in nutrients, while others proposed that these were features of FOF.

There was also confusion about when specialist formula might be needed (theme 2.3). Some viewed products labelled as ‘Comfort milk’ or ‘lactose-free’ as a discrete category of specialist products for babies with medical issues. For others, brand eclipsed all other labelling and specialist formulas were viewed as similar to the brand’s other products. A lactose-free formula was seen by one mother as a good choice as it was labelled as a ‘breastmilk substitute’ which indicated to her a greater similarity to breast milk. Comfort formula (marketed for colic and constipation) was used by one mother because her baby experienced reflux and another believed it was intended for keeping babies fuller for longer. Specialist formulas were generally used without consulting a health professional. Many felt specialist formulas should include a clearer explanation on the front of the pack.

### Theme 3. Presentation matters

The appearance of packs, including colours and images, was fundamental to mothers’ understanding of the differences between products (theme 3.1). Products with primary colours and pictures of animals and teddy bears attracted some, who described them as warmer, more child-friendly and less overwhelming. Others felt these looked cheap, preferring products with silver packaging and science-related imagery, which they described as more professional, ‘medical-y’ or ‘science-y’. Mothers were aware that this latter group of products was more expensive, which some took as another indicator that they were better, although they were unsure how (theme 3.2). Others suspected price just reflected additional marketing and one wondered if they might just be choosing between different types of packaging.

Mothers described different messaging features being particularly salient (theme 3.3). Products labelled as organic with accompanying rural imagery were described as more natural, more nutrient rich, healthier, having ‘more properties’ or ‘just better’. A small number of mothers reported being attracted to their current formula because it was marketed as palm oil free, despite not previously considering palm oil or knowing whether it was included in other CMF. Nutrient claims were generally disregarded but the nutrient content panel was seen as reassuring although most participants reported not really understanding it.

Messaging about science, expertise and research provided a sense of confidence in products. The phrase ‘nutritionally complete’ was perceived to mean a product contained everything babies need, indicating it was equivalent to breast milk. One brand’s logo was interpreted by most mothers as representing a mother breast feeding, suggesting greater similarity to breast milk than other products.

## Discussion

This qualitative study explored how mothers use CMF labels when choosing products and how they understand differences between products. Legislation focuses on nutrition and health claims as promotional tools but mothers in the current study paid little attention to these. Instead, mothers reported the brand name on packs to be key to determining choice. On-pack messaging interpreted as indicating superiority of one product over another and greater similarity to breast milk was also salient. These findings provide novel first-hand insights into the dynamic and covert ways in which CMF marketing influences maternal feeding choices.

Mothers described being drawn to ‘big brands’ which they were familiar with long before having a baby. This tallies with WHO’s concerns over the pervasive and invasive nature of formula marketing which successfully constructs reassuringly familiar and evocative brands.^12 19^ It also provides evidence to support calls for marketing loopholes to be closed as the current practice of allowing FOF and GUF to be advertised in near identical packaging is clearly informing IF choices in the first days and weeks of infants’ lives.^9^

Participants assumed that as multiple brands and product lines are available, they must be substantially different. In line with previous studies, caregivers were confused about these differences.^20^ Despite scrutinising labels, many felt a sense of failure at being unable to identify ‘the best one’, indicating that current labels simply add to mothers’ anxiety or confusion, rather than facilitating informed choice. As in previous research, mothers believed that if FOF and GUF were not necessary they would not exist.^26^ For some, this went against advice from health professionals to continue with IF then transition to cow’s milk at 12 months, creating uncertainty and self-doubt. The perceived need for GUF is problematic as they are expensive ultra-processed foods high in free sugar, which can have a substantial effect on family food budgets.^27^ Confusion over the use of specialist formula also adds to concerns that products are frequently misused and hence should not be available over the counter.^28^

References to science and nature were salient, and labelling products as organic or palm oil free had a powerful health halo effect, giving the impression that products were healthier, more natural and more similar to breast milk. This echoes findings that images and health halo statements, rather than overt health and nutrition claims, influence how healthy parents perceive children’s food to be.^29^ In line with Hastings *et al*’s description of targeted marketing, we found on-pack references to science and research attracted some mothers, while others found child-friendly packaging more appealing.^12^ The use of messaging that confuses caregivers about similarities, such as referencing breast milk and IF in the same sentence, has previously been described.^30 31^ In line with this, we found a logo perceived as resembling a breastfeeding mother, references to nature and the words ‘breastmilk substitute’ were interpreted as suggesting that some products were more similar to or equivalent to breast milk.

HCPs can play a powerful role in countering commercial pressure to buy more expensive CMF. Our findings highlight the need for HCPs to help parents navigate the CMF market. Advising parents to choose ‘the best one for you’ drove some to seek advice from companies’ websites. Whereas when HCPs informed mothers that only IF was recommended and that expensive products were no better than cheaper ones, this relieved parental anxiety and facilitated more informed choice. This clear advice should be routinely communicated, although ultimately, only legislative change will ensure caregivers receive consistent information about feeding.

Our findings provide evidence showing labelling legislation should be tightened to more effectively counter persuasive implied claims. Although we focused on labelling, television and digital marketing were repeatedly referenced by mothers, which supports calls for stronger CMF policies and monitoring systems more generally.^8 15 32^

Fathers, other caregivers and society also play a crucial role in shaping infant feeding norms and it is a limitation of this study that only mothers took part. The number of participants was also relatively small and most were recruited via Facebook which may have impacted representativeness, but it is a strength of the study that mothers from diverse backgrounds were included. The effect of collecting data during the COVID-19 pandemic is unclear.

## Conclusions

Loopholes in CMF legislation should be tightened and enforced to facilitate informed choice about infant feeding. This includes restricting communication channels creating brand familiarity and removing images and messaging on labels that are perceived as suggesting certain CMF products are superior to others or that some products have a greater similarity to breast milk.

## Data Availability

No data are available. To ensure the anonymity of participants, transcripts are not available.
