# Supplementary material for: Perceived influence of commercial milk formula labelling on mothers’ feeding choices in Great Britain: a qualitative study
Source: Arch Dis Child. 2023 Aug 4;108(12):1008–13. doi: 10.1136/archdischild-2023-325767 (PMC10715500; doi:10.1136/archdischild-2023-325767)
Supplement: Supplementary data [file archdischild-2023-325767supp001.pdf]

**Infant Feeding Choices Study Interview Schedule**

**Part A. Explore personal formula and feeding choices**

1. **First of all can you tell me what your daughter's/son's favourite foods are at the moment?**

2. **Can you tell me about how you've fed her/him since he/she was born?**
(1. breastfeeding/**formula** - details of different formulas used, specialist milks, brands of formula. 2. **Food** – homemade & commercial, number of meals & snacks)

3. **Can you talk me through how you chose this/each particular formula?**
(brand, product range, stage, specialist milk. Why switched**. Looking for anything in particular from a product?** (e.g. nutrients, health benefits, price, organic, anti-reflux)

4. **If you can think right back to when you were pregnant, can you tell me what your thoughts were about how you might feed?** (some pp have said they felt a pressure to breastfeed or bottle feed – how was that for you?)

5. **Did you get information from anywhere or anyone that helped you decide which formula to choose?** (e.g. midwife, health visitor, bounty packs, leaflets, TV adverts, magazines, online (websites visited eg formula brand/NHS), baby groups, family, friends)

**Part B. Explore understanding of formulas available**

On the screen are some formula milks that are available now. What I'd like you to do is move them around and put them into groups depending on what you might think of as similar products. You can make as many groups as you like and feel free to move them around and re-organise them as you like.

If you could talk me through what you're thinking about while you're doing this that would be really helpful.

1. **What are your first thoughts that come to mind? What is it about these that makes them seem similar?**
2. 
3. **Some of the products have numbers on, so can do you tell me how does it all work?** How are the milks different? How important is it for babies to have these different milks at particular ages or stages?

4. **Some products are more expensive, why do you think that is?** Are these milks different?

5. **Looking at the information and the look of the packs. What do you notice/feel is important?**

**Part C. Thoughts about particular features on formula packs**

There's lots of information shown on formula labels and I've just got one here that I'd like you to look at more closely.

1. **Which parts of pack might you usually look at?**

2. **Can you tell me about the information on each side and what you think about it?**
   **How would you describe the images?**
   **What does that inform you about giving this product to your baby rather than another one?**

3. **How does that experience of looking closely at pack compare to what you've done before when choosing?**

4. **What other information might you like to see on a label or how might it be arranged differently to help you make a decision about which one to buy?**
